# Selection and preliminary application of a single stranded DNA aptamer targeting colorectal cancer serum†

**DOI:** 10.1039/c9ra04777h

**Published:** 2019-11-27

**Authors:** Kun Li, Liqing Qi, LiMing Gao, Ming Shi, Jian Li, ZhiWei Liu, Lu Zhao

**Affiliations:** College of Environmental & Chemical Engineering, Yanshan University Qinhuangdao Hebei Province 066004 China likun@ysu.edu.cn; Applied Chemistry Key Laboratory of Hebei Province, Yanshan University Qinhuangdao Hebei Province 066004 China; Engineering Research Center of Functional Nucleic Acids in Qinhuangdao Qinhuangdao Hebei Province 066004 China; The First Hospital of Qinhuangdao City Qinhuangdao 066000 China

## Abstract

Colorectal cancer is one of the common causes of malignant tumors in recent years, thus the discovery of potential compounds that detect the occurrence of colorectal cancer by efficient approaches is necessary. In this study, the method of systematic evolution of ligands by exponential enrichment (SELEX) was used for recognizing serum from colorectal cancer patients by a single-stranded DNA library of aptamers assisted by single-walled carbon nanotubes (SWCNTs) to remove single-stranded DNA with low affinity. Ten rounds of selection were applied using colorectal cancer serum as a target with the serum of healthy individuals as a control. As the result, we have successfully identified four candidate aptamers after high-throughput genome sequencing analysis, comparison analysis and secondary structure prediction. Among them, aptamer Seq-2 exhibited the highest affinity and the strongest selectivity with an equilibrium dissociation constant (*K*_d_) of 11.31 ± 3.25 nM and a *C*_t_ difference value of 4.25 ± 0.38 between the colorectal cancer group and the healthy group. Moreover, with fifty negative control serum samples, the positive detection rate of fifty positive serum samples tested by aptamer Seq-2 was over 90%. In particular, aptamer Seq-2 can strongly bind the colorectal cancer serum, less strongly bind the non-colon cancer serum and hardly bind the healthy serum. Therefore, aptamer Seq-2 presents enormous potential for exploring as a tumor diagnostic kit and detecting unknown tumor markers in serum to reflect colorectal cancer.

## Abstract 2

Aptamer Seq-2 with high affinity and selectivity was screened against colorectal cancer serum directly for clinical application.

## Graphical abstract

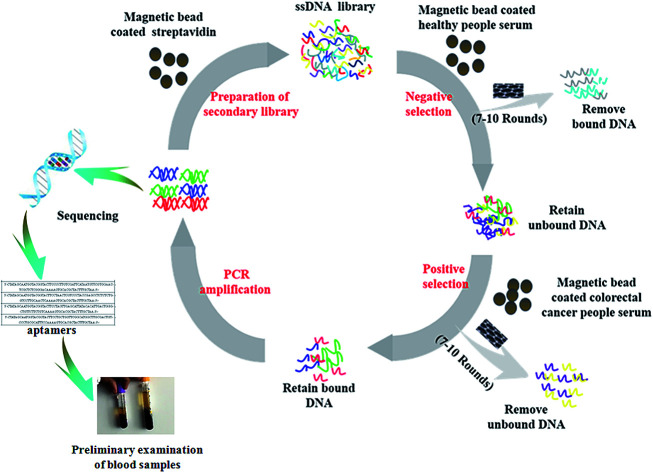

## 1. Introduction

Colorectal cancer, as a common cause of malignancy in the digestive tract, can occur anywhere in the colon. Colorectal cancer has relatively high incidence in North America and Western Europe, and its mortality ranks 2nd of all the malignant cancers in the United States. Meanwhile, its mortality ranks 5th or 6th of all the malignant cancers in most provinces in China, and keeps going up in recent years.^1,2^ Therefore, early diagnosis and treatment is the key to improve the survival rate of patients with colorectal cancer.^3^ At present, the early diagnosis of colorectal cancer depends mainly on endoscopies and tumor markers in the serum. Given that serum is the best specimen for *in vitro* diagnosis of cancer, detection of tumor markers in the serum is a relatively good, non-invasive way for clinical diagnosis of colorectal cancer.^4^

Nucleic acid aptamers, single stranded random oligonucleotide library artificially synthesized through ligand evolution technique (SELEX) in exponential amplification,^5,6^ can be used as one of the most promising tools for disease diagnosis due to their high specific affinity to targeted molecules.^7^ They are also objectively measurable molecular signatures of physiological status that can be used as disease indicators or drug targets for clinical diagnosis,^8,9^ imaging,^10,11^ delivery^11,12^ and therapy,^13–15^ hence playing a role in precision medicine. There are already many successful studies about aptamer-mediated gene therapy on breast cancer, glioma, prostate cancer, Burkitt lymphoma, *etc.*^16,17^, but the application of aptamers as tools for diagnosis of cancer is rarely reported because of its natural characteristics. Aptamers can bound a large-scale of targets including proteins,^4,18^ peptides,^19^ nucleotides,^20^ antibiotics,^21^ toxins^22^ and small molecules^23^ all with high affinity and selectivity. However, serum-targeted screening is more complex and difficult than ever.

Although the serum can be applied as the best specimen for *in vitro* diagnosis of cancer, tumor markers in the serum of the colorectal cancer patients were mainly recognized by antibodies up to date.^24,25^ However, there are some drawbacks such as complicated synthesis route, high cost, relatively weak selectivity and affinity for the antibodies. And the relatively weak selectivity and affinity leads to low accuracy to recognize colorectal cancer. Moreover, extraction of tumor markers from the serum of the colorectal cancer patients is relatively complicated, leading to few tumor markers available for clinical diagnosis and to the reduction in recognition efficiency of colorectal cancer.^26^ Therefore, there is still a troublesome issue to develop some simple diagnosis tools with high accuracy to recognize colorectal cancer based on the serum of the colorectal cancer patients.^27–29^

In this study, we have developed an efficient strategy to screen a highly specific nucleic acid aptamer aiming to recognize colorectal cancer serum as diagnosis tools.^30–38^ Firstly, high abundance proteins in the serum could be efficiently removed using acetonitrile as a precipitate to reduce the enrichment of nonspecific ssDNA libraries, thus improving the screening efficiency of the final aptamers. Secondly, during the successive positive-screening and negative-screening processes, the mixture of the high abundance proteins-removed serum and primary ssDNA libraries with the carboxyl magnetic agarose beads as separation medium was treated through successive incubation, elution and thermal denaturation steps to collect the primary ssDNA libraries with highly specific affinity to tumor markers in the serum of the colorectal cancer patients. In the elution steps of latter screening, the carbon nanotubes were added to the washing solution to remove ssDNA with low affinity thereby improving the efficiency of screening. Thirdly, the obtained primary ssDNA libraries were amplified by a PCR technique to avidin-containing dsDNA sequences, and avidin-containing dsDNA sequences with magnetic streptavidin beads as separation medium were treated successive incubation, elution and thermal denaturation steps to prepare the secondary ssDNA sequences for the next round of screening.

After 10 rounds of screening, we obtained the candidate aptamers with highly specific affinity to tumor markers in the serum of the colorectal cancer patients. Just as we expected the generated aptamers were proved possessing high affinity and strong selectivity against target serum. With fifty serum samples from healthy persons as the negative control samples, all the positive rates of fifty serum samples from the colorectal cancer patients using these four enriched aptamers as the probe molecules are all over 80%, attributing to the highly specific affinity of these aptamers to unknown tumor markers in the serum of the colorectal cancer patients. Prospectively, generated aptamer Seq-2 can directly diagnose colorectal cancer through the serum samples of colorectal cancer patients instead of tumor markers extracted from the serum samples of colorectal cancer patients, dramatically enhancing recognition efficiency of colorectal cancer. Therefore, *in vivo* study indicated that aptamer Seq-2 could be a potential molecular probe for colorectal cancer diagnosis and further research.

## 2. Materials and methods

### 2.1 Materials

Random ssDNA library: 5′-CTATAGCAATGGTACGGTACTTCC-(40N)-CAAAAGTGCACGCTACTTTGCTAA-3′, P6 primer (5′-CTATAGCAATGGTACGGTACTTCC-3′), P9 primer (5′-TTAGCAAAGTAGCGTGCACTTTTG-3′), and P9 (biotin) primer (5′-Biotin-TTAGCAAAGTAGCGTGCACTTTTG-3′) are purchased from TaKaRa. Carboxyl agarose beads is obtained from Beyotime Biotechnology (Shanghai, China) Co., Ltd. The serum samples of both colorectal cancer patients and healthy persons are gained from Qinhuangdao First Hospital, Hebei province in China. SWCNT is purchased from Tanfeng Tech. Inc (Suzhou, China). Ultrafiltration centrifuge tubes (3KD) is provided by Millipore (Beijing, China). All related PCR reagents are acquired from Sangon Biotech (Shanghai) Co., Ltd. today Mai.

### 2.2 Treatment of blood samples

The whole blood samples of twenty colorectal cancer patients were collected, and then centrifuged at 4000*g* for 10 min at room temperature to remove red blood cells. Subsequently, the collected serum samples were centrifuged at 15 000*g* for 30 min at 4 °C to remove surface grease in the upper layer and precipitate in the lower layer and collect the solution in the middle layer. The collected solution was stored at −80 °C for further use. The whole blood samples of twenty healthy persons were treated in the same method to the treatment of the whole blood samples of colorectal cancer patients mentioned above. All these experiments were performed in compliance with the relevant laws and institutional guidelines of China and the ethics committee that approved these experiments was the Review Board of the First Hospital of Qinhuangdao City. Informed consent was also given by every participant.

### 2.3 Removal of high abundance proteins in serum from colorectal cancer patients and healthy persons

The serum samples of twenty colorectal cancer patients were fully mixed with autoclaved ultrapure water and acetonitrile at a volume ratio of serum : autoclaved ultrapure water : acetonitrile of 1 : 2 : 0.5, followed by ultrasonication for 5 min. Then, the solution was centrifuged at 15 000*g* for 30 min to collect the supernatant liquid for further use. The serum samples of healthy persons were treated in a similar method.

### 2.4 Negative screening

The mixture of the high abundance proteins-removed serum of healthy persons (1 mL) and PBS solution (4 mL, pH 7.4) were fully mixed with the carboxyl agarose beads (4 mL, 1 mg mL^−1^) into a EP tube and then incubated at 37 °C for 2 h. Subsequently, the product in the negative tube was magnetically recovered, and rinsed three times with PBS solution (3 min/time). The magnetically recovered agarose beads were dispersed into the 4 mL blocking buffer (1.25 g L^−1^ BSA, 0.1 g L^−1^ yeast tRNA, 0.1 g L^−1^ salmon sperm DNA, 137 mM NaCl, 2.7 mM KCl, 10 mM Na_2_HPO_4_, 2 mM KH_2_PO_4_, pH 7.4) to incubate at 37 °C for 1 h followed by the addition of the initial single-stranded DNA library incubated at 37 °C for 1 h again. The supernatants containing unbound ssDNA library were collected for further use. The magnetically recovered agarose beads in the negative sieve tube were douched three times with washing buffer and once with ultrapure water. After the agarose beads in the positive sieve tubes being mixed with ultrapure water (2 mL), the mixture was incubated for 5 min at 95 °C and centrifuged at 13 500*g* for 2 min to collect the supernatant solution (2 mL). The treatment of the agarose beads in the positive tubes with autoclaved ultrapure water (2 mL) repeated successively twice to collect 6 mL of the total supernatant solution then added in ultrafiltration centrifuge tubes to centrifuge (8000 rpm) until remaining 500 μL of liquid for detection by RT-PCR.

### 2.5 Positive screening

The mixture of the high abundance proteins-removed serum of colorectal cancer patients (1 mL) and PBS solution (4 mL) at a pH value of 7.4 were fully mixed with carboxyl agarose beads (4 mL) into a EP tube and then incubated at 37 °C for 2 h. Subsequently, the product in the positive tube was magnetically recovered, and rinsed three times with PBS solution (3 min/time). The magnetically recovered agarose beads were dispersed into the 4 mL blocking buffer (1.25 g L^−1^ BSA, 0.1 g L^−1^ yeast tRNA, 0.1 g L^−1^ salmon sperm DNA, 137 mM NaCl, 2.7 mM KCl, 10 mM Na_2_HPO_4_, 2 mM KH_2_PO_4_, pH 7.4) and the mixture was incubated for 1 h at 37 °C. The magnetically recovered agarose beads were washed with PBS solution three times, followed by the addition of the supernatants containing unbound ssDNA collected in negative screening. After the above mixture being incubated at 37 °C for 1 h, the agarose beads were recovered. The magnetically recovered agarose beads in the positive sieve tube were douched three times with washing buffer and once with ultrapure water. The agarose beads were successively treated with autoclaved ultrapure water (2 mL) three times and then to centrifuge according to a method similar to the treatment of the beads in the positive sieve tube, finally 500 μL of the remaining supernatant solution was obtained for RT-PCR detection and amplification used preparation of secondary ssDNA library.

### 2.6 Preparation of secondary ssDNA library through amplification of RT-PCR and monitoring of enrichment effects

2× SG Fast qPCR Master Mix (120 mL), P6 primer (10 μM, 4.8 μL), P9 (biotin) primer (10 μM, 4.8 μL), DNF Buffer (24 μL), PCR-grade water (38.4 μL) and the recovered supernatant solution (48 μL) in the positive sieve tube were mixed thoroughly. And the above homogeneous solution was packed equally in eight tubes to accomplish PCR amplification of ssDNA library until its amplification reaching the maximum of the S curve. The amplified product was mixed thoroughly with streptavidin-labelled magnetic beads (0.3 mL), and the mixture was incubated at 37 °C for 40 min. Subsequently, the magnetically recovered beads was dispersed into PBS solution (1 mL) containing 1% Tween-20 solution and incubated at 40 °C for 5 min, and the treatment of the magnetically recovered beads with a 1% Tween-20 solution (1 mL) repeated three times. Finally, the magnetically recovered beads were homogeneously dispersed into ultrapure water (200 μL) and the mixture was incubated at 95 °C for 5 min to collect the supernatant as the secondary ssDNA library for the next round of screening.

In this way, negative screening and positive screening were performed multiple times. For increasing the selection pressure during the screening process, SWCNT are introduced to be added to the washing solution to compete with the target from the seventh round to help improve efficiency by improving the concentration of SWCNT and the strength of the washing solution so that remove some single-stranded DNA with low affinity to the target serum to a greater extent. In each round of screening, ssDNA libraries binding to the serum of colorectal cancer patients and to that of healthy persons were collected, and their enrichment extents were monitored through real-time quantitative PCR technique. The purity of the amplified product was observed by gel imaging of agarose gel electrophoresis.

### 2.7 The assessment of the affinity and selectivity of the candidate aptamers by multimode microplate reader

The ssDNA library obtained from the tenth round of screening was delivered to Sangon Biotech (Shanghai) Co., Ltd. to perform high throughput sequencing analysis. The aptamer candidates were grouped based on their sequential homogeneity and 4 representative sequences from diverse groups were chosen and synthesized by Sangon Biotech (Shanghai) Co., Ltd.

Subsequently the binding affinities of these 4 candidate aptamers were determined by incubating various concentrations of FAM labeled sequences and FAM-labeled random ssDNA with the beads-serum at 37 °C overnight. After incubation, the beads in the EP tubes were washed three times with 500 μL washing buffer and mean fluorescence intensity was determined by multimode microplate reader. The ligand binding analysis function of Origin7.5 was used to calculate the apparent *K*_d_ of aptamers according to the equation *Y* = (*B*_max_ × *X*)/(*K*_d_ + *X*) (where *Y* is the net fluorescence intensity at each concentration, the *B*_max_ is the saturated binding and the *X* is the concentration of aptamer in nM).

For the assessment of the selectivity of the candidate aptamers by multimode microplate reader, two sets of experiments were carried out. Some FAM-labeled aptamer pools (300 nM) including the four candidate aptamer sequences were incubated with colorectal cancer serum (50 μL) and the four candidate aptamer sequences (300 nM) were tested with multiple serum proteins (50 μL) followed with determination of fluorescence intensity by multimode microplate reader (SpectraMAX M2e, USA).

### 2.8 Selectivity of aptamer candidates by PCR

Each serum sample (50 μL) from colorectal cancer patients, agarose beads (50 μL), and PBS solution containing each candidate aptamer were mixed, further the corresponding mixture was incubated at 37 °C for 2 h. After incubation, the beads were washed three times with 1 mL 0.01% PBS. For blocking, each tube was added with 200 μL of 3% BSA in PBS and was then incubated at 37 °C for 2 h. One hundred microliters of aptamer candidate pools (300 nM) was added for 1.5 h at 37 °C. Subsequently, the recovered agarose beads were washed with PBS solution containing a 0.1% Tween-20 solution five times and with autoclaved ultrapure water once. Finally, the recovered beads were dispersed into autoclaved ultrapure water (20 μL), and the mixture was treated at 95 °C for 5 min to collect the supernatant dissolved the recovery ssDNA. The serum samples from healthy persons were used as negative control samples to evaluate recognition accuracy of colorectal cancer with our four aptamers as probes. Each serum sample from healthy persons was treated through the similar method to the treatment of serum sample from colorectal cancer patients. We also did the blank control with only alternation that autoclaved ultrapure water were used to replace serum samples to make sure that the removal of some ssDNA binding with other substances but for the serum samples such as BSA in blocking solution. Finally we monitored the selectivity according to the Δ*C*_t_ (the difference value between the *C*_t_ value of the collected ssDNA binding to the serum from colorectal cancer patients and the collected ssDNA binding to the serum from the healthy people) value through real-time quantitative PCR technique.

### 2.9 Detection of blood samples with a molecular tool of aptamer Seq-2

To perform the aptamer-based detection of colorectal cancer assay by human blood samples, 50 μL agarose beads with 50 μL serum were incubated at 37 °C overnight, followed by washing three times with 200 μL washing buffer. The obtained beads were then blocked by 200 μL of 3% BSA in the washing buffer and washed three times with washing buffer. After that, 100 μL aptamer FAM-labeled Seq-2 (300 nM), which has the highest affinity and strongest selectivity of was added and incubated at 37 °C for 1.5 h. After washing three times with washing buffer containing 0.1% (wt) Tween-20 solution, the beads were finally suspended in 200 μL Milli-Q water. The luminescence value was measured by multimode microplate reader (SpectraMAX M2e, USA) at 520 nm under 25 °C. Next we recycled these beads back into the corresponding EP tubes and removed as much water as possible from the EP tubes, followed by adding 20 μL Milli-Q water to each tubes. Finally, the mixture was treated at 95 °C for 5 min to collect the supernatant dissolved the recovery ssDNA to monitored the selectivity according to the Δ*C*_t_ value through real-time quantitative PCR technique as mentioned above.

We tested blood samples from 25 patients with colorectal cancer and 25 healthy persons totally, and each case was done in parallel.

### 2.10 Statistical analysis

Graphpad Prism 5.0 were used for data analysis. Values of interest were presented as the mean plus or minus standard deviation. Statistical analysis was performed using the Student's *t*-test methods. Differences were considered statistically significant if *P* value < 0.05.

## 3. Results

### 3.1 Generation of nucleic acid aptamers against the serum of the colorectal cancer patients

**Fig. 1:**
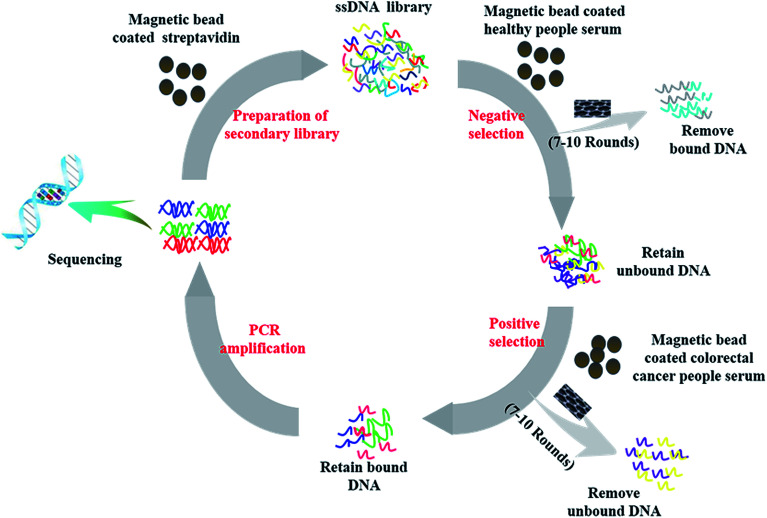
Schematic illustration of a subtractive SELEX protocol for generation of aptamers toward colorectal cancer serum.

Fig. 1 showed a summary of the selection process of each round, which mainly consisted of four steps including negative selection, positive selection, PCR amplification and the preparation of the secondary ssDNA library. The initial ssDNA libraries contained 10^16^ unique sequences for rich sequence diversity. Furthermore, we performed changes a serious important parameters involved in the screening process (as shown in Table S1†) to increase selection pressure. The success of screening is directly related to the amounts of ssDNA of each round of screening. Therefore, in the initial rounds of screening, a large amount of ssDNA library and the serum samples from colorectal cancer patients were used in order to obtain specific ssDNA libraries for the serum samples mentioned above. In the subsequent rounds, the aim of screening is to screen nucleic acid aptamers with higher selectivity and stronger affinity to the serum of colorectal cancer patients than other ssDNA libraries. Accordingly, the amounts of both ssDNA library and the serum samples from colorectal cancer patient are decreased and their volume ratio is increased and SWNCT were added to washing buffer later, which ensures that ssDNA libraries with stronger affinity to probable tumor markers in the serum of colorectal cancer patients than other ssDNA libraries that could be screened. Finally, we obtained four candidate nucleic acid aptamers (as shown in Table 1) with high affinity and strong selectivity through ten rounds of SELEX screening technique.

**Table 1:** Sequences of candidate nucleic acid aptamers against the serum of colorectal cancer patients

Aptamer name	Sequence
Seq-1	5′-CTATAGCAATGGTACGGTACTTCCCCTTGTCGATTCATAATGTTCGTGGAACTCGCTCTCGGGACAAAAGTGCACGCTACTTTGCTAA-3′
Seq-2	5′-CTATAGCAATGGTACGGTACTTCCTAACTCGTCCCTACCGAGCCTCTCTCTGGTCCTTGCAACTCAAAAGTGCACGCTACTTTGCTAA-3′
Seq-3	5′-CTATAGCAATGGTACGGTACTTCCTAGTTGAGCATTATACACATTGACTGGGCTGTTCTTCTGTCAAAAGTGCACGCTACTTTGCTAA-3′
Seq-4	5′-CTATAGCAATGGTACGGTACTTCCTGCTGGTTCGGCATGGCTTGCGACTGTCCCTGCGCATTTCCAAAAGTGCACGCTACTTTGCTAA-3′

As stated in the Fig. S1,† the aptamer binding rates of the serum from colorectal cancer patients can be calculated according to the following ratio: binding rates (%) = ssDNA_recovered_/ssDNA_initial_ × 100. Among them, ssDNA_recovered_ refered to the amount of the recovered ssDNA library and ssDNA_initial_ referred to the amount of the initial ssDNA library, those amounts were all listed in the Table 1. We calculated that binding rate of the aptamers targeting the serum of colorectal cancer patients after the first round of screening was merely 1.66%. With the numbers of rounds increasing, the aptamer binding rate increased gradually. This rate reached 5.47% after the fifth round of screening, and went up to the maximum value of 19.6% after the ninth round of screening. Compared to the aptamer binding rate after the ninth round of screening, the aptamer binding rate after the tenth round of screening showed no obvious changes, which demonstrated that the aptamer binding rate after ten rounds of screening had reached saturation without extra rounds of screening. Accordingly, nucleic acid aptamers with high affinity and selectivity for the serum of colorectal cancer patients were finally screened out. Gel imaging by agarose electrophoresis of the 10th round of ssDNA amplification products (Fig. S2†) revealed a clear single band having a molecular weight of 88 bp similar with random ssDNA. Furthermore, we chose the ssDNA library after the tenth round to carry out high-throughput sequencing in Sangon Biotech (Shanghai, China) Co., Ltd. Finally, we performed a series of sequence analysis and acquired four candidate aptamers (aptamer candidates include the randomized region plus primer binding sites) for affinity and selectivity evaluation and other further studies which were listed in Table 1. Four screened aptamer candidates for the serum of colorectal cancer patients all have the same length. In these four sequences, their two lateral fixed sequences are obtained as designed, and their middle random sequences do not own common conservative sequence.

### 3.2 Secondary structure prediction of the four candidate aptamers for the serum of colorectal cancer patients

These four candidate aptamers not only showed high repetition frequencies in the aptamer pools, but also contained high GC contents, which suggested a secondary structure formed. The enrichment of these four sequences with secondary structure motifs in the aptamer pool reflects the preferential selection for these structures as a result of SELEX. Generally the most favored aptamer structure were with the highest affinity because of a serious of important parameters being changed in the whole screening process to increase selection stringency.^49^

The secondary structures of the four candidate aptamers for the serum of colorectal cancer patients were predicted in Fig. S3† through IDT (Integrated DNA Technologies) based on the principle the lowest energy for these aptamers folding into complex and stabling three-dimensional shapes enabled them to bind to target molecules. All the second structural features of the four oligonucleotide aptamers mainly consists of stem rings and convex rings. It is speculated that the existence of these stem rings and convex rings structures may be the structural basis for the highly specific binding of nucleic acid aptamers serum targets from colorectal cancer patients. Presumably the stem in the stem-ring structures may serve the function of stabilizing the targets, and the rings may bind to the targets.

### 3.3 The assessment results of the affinity and selectivity of the candidate aptamers by multimode microplate reader

**Fig. 2:**
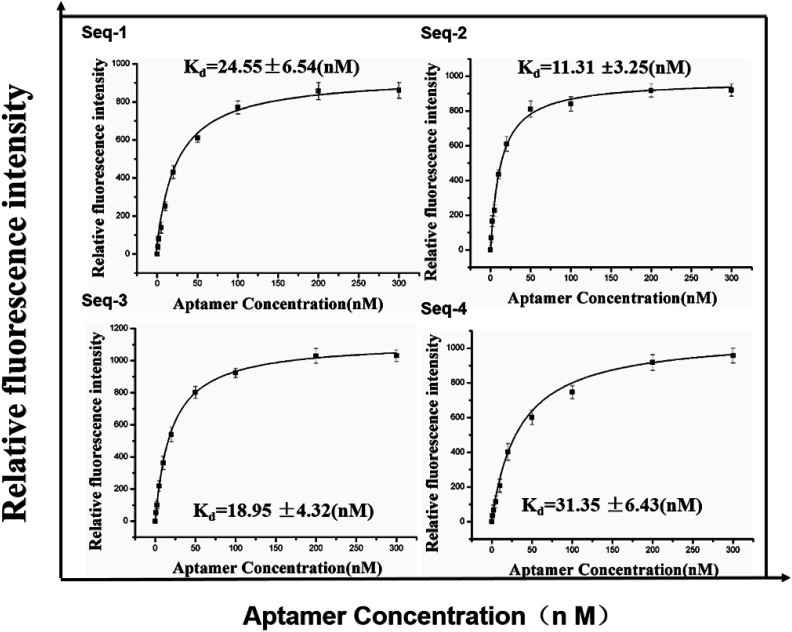
Binding saturation curve of four aptamer candidates to colorectal cancer serum. Error bars represent standard deviation from triplicate analysis.

To investigate the binding affinity to the serum of colorectal cancer patients, we evaluated it by incubating these FAM-labeled aptamer candidates of different concentrations with constant volume of positive target serum with FAM-labeled random ssDNA library as contrast. The fluorescence spectrum of the mixture were detected with a fluorescence spectrometer with excitation at 490 nm. Subsequently, we further estimated the equilibrium dissociation constants (*K*_d_) from binding saturation curve of four aptamer candidates to colorectal cancer serum (Fig. 2) and explained favorably that the all aptamer candidates showed high binding ability in the submicromolar range against the serum of colorectal cancer patients.

**Fig. 3:**
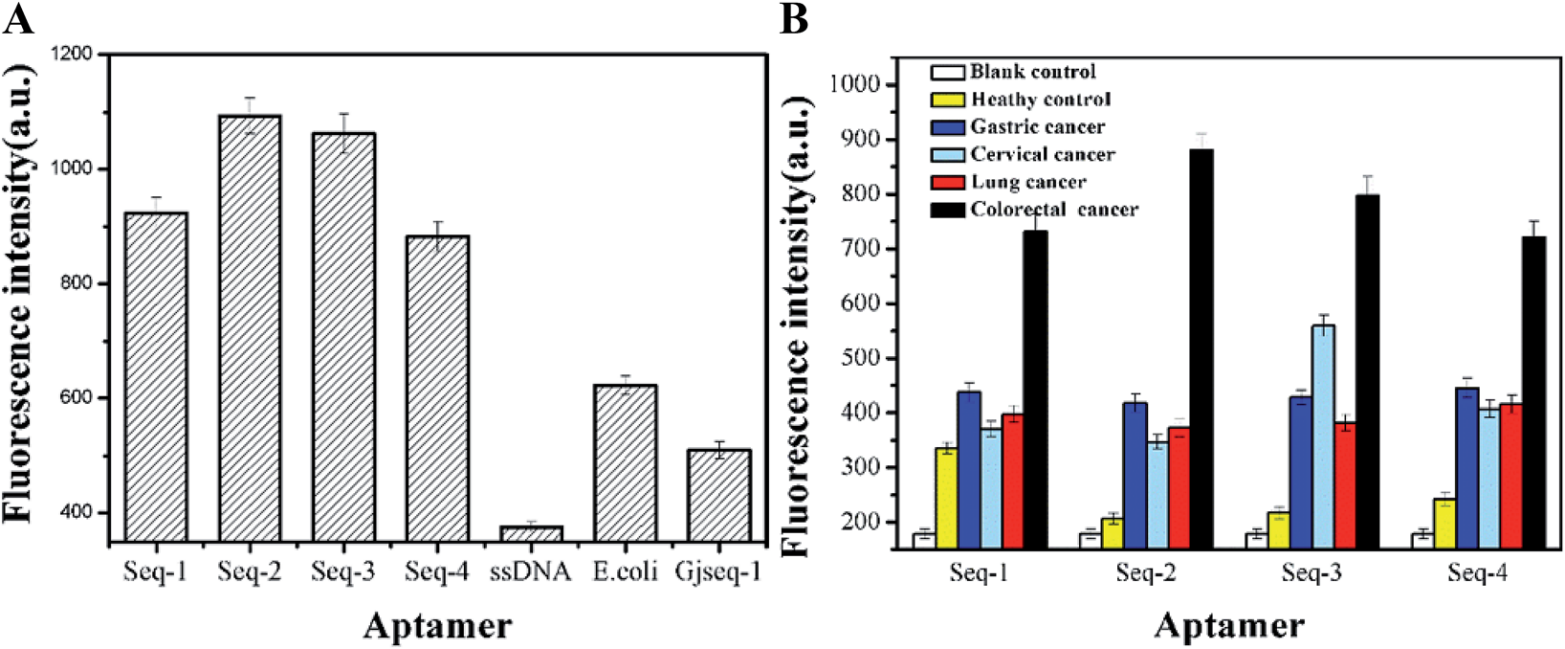
Selectivity analysis of aptamer candidates based fluorescence. (A) the evaluation the selectivity of these aptamer sequences with three control aptamer pools toward colorectal cancer serum. (B) The four aptamer candidates tested with multifarious serum proteins. Error bars represent standard deviation from triplicate analysis.

To ensure that the candidate aptamers are specific to serum of colorectal cancer patients, we performed two sets of experiments based fluorescence method to verify their selectivity. From the results of the evaluation the selectivity of these aptamer sequences with three control aptamer pools (random-ssDNA, a anti-*Escherichia coli* aptamer and a anti-cervical cancer serum aptamer Gjseq-1 studied by our laboratory which the sequences have not published) toward colorectal cancer (Fig. 3A) and the four aptamer candidates tested with multifarious serum (Fig. 3B), although generally lower fluorescence value, we can obtain the information that the four aptamer candidates are able to distinguish colorectal cancer serum with other cancer serum and healthy human serum and the aptamer Seq-2 is the best.

### 3.4 Selectivity analysis of aptamer candidates using quantitative real-time PCR

**Fig. 4:**
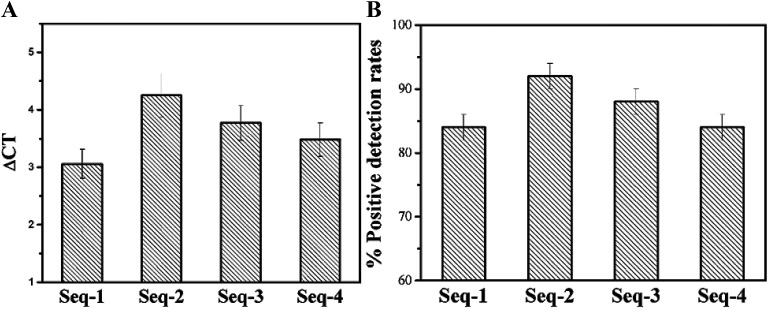
Selectivity analysis of aptamer candidates by PCR. (A) Mean ΔCT of the four aptamers against 50 serum samples from colorectal cancer patients' samples. (B) The positive detection rates of the four aptamers against 50 serum samples from colorectal cancer patients samples. Error bars represent standard deviation from 50 serum samples analysis.

To further verify the selectivity of candidate aptamers, we also performed a series of PCR-based experiments. With fifty serum samples from healthy persons as the negative control samples, the *C*_t_ value of fifty serum samples from colorectal patients were measured by PCR after calculating the all ΔCT values (as shown in Fig. 4A).

Inevitably, samples with failed tests were appeared so that we also calculated the positive detection rate of the four aptamer candidates. All the ΔCT values displayed positive of the four candidate aptamers were between 2.5 and 5 cycles, which means that all the four candidate aptamers are almost exclusively combined with the serum of colorectal cancer patients, and are not combined with the serum of healthy persons without colorectal cancer (as shown in Fig. 4A). As expected, with fifty serum samples from healthy persons being the control samples, all the positive detection rates of fifty serum samples from the colorectal cancer patients using these four enriched aptamers as the probes are all over 80% which further illustrate the highly specific affinity of these aptamers to unknown tumor markers in the serum of the colorectal cancer patients (Fig. 4B). Delightfully the mean ΔCT values come up to 4.25 and the positive detection rate reaches 92.0 ± 2.7% of Seq-2 nucleic acid aptamers. The results consistent with the method based fluorescence show that Seq-2 is the best.

### 3.5 Biorecognition with aptamer Seq-2 for blood samples of human

According to the *K*_d_ values, predicted secondary structures and identification results, we chose Seq-2 aptamer as a molecular tool to detect natural blood samples directly in practical applications. To validate the feasibility, serum of colorectal cancer patients and healthy human was monitored with FAM-labeled Seq-2 aptamer. Then we read the fluorescence value by a multi-function microplate reader (SpectraMAX M2e, USA). Moreover, as a further study to confirm the reliability of Seq-2, we calculated the CT values with PCR by recovering the ssDNA binding chemically on the magnetic beads.

**Fig. 5:**
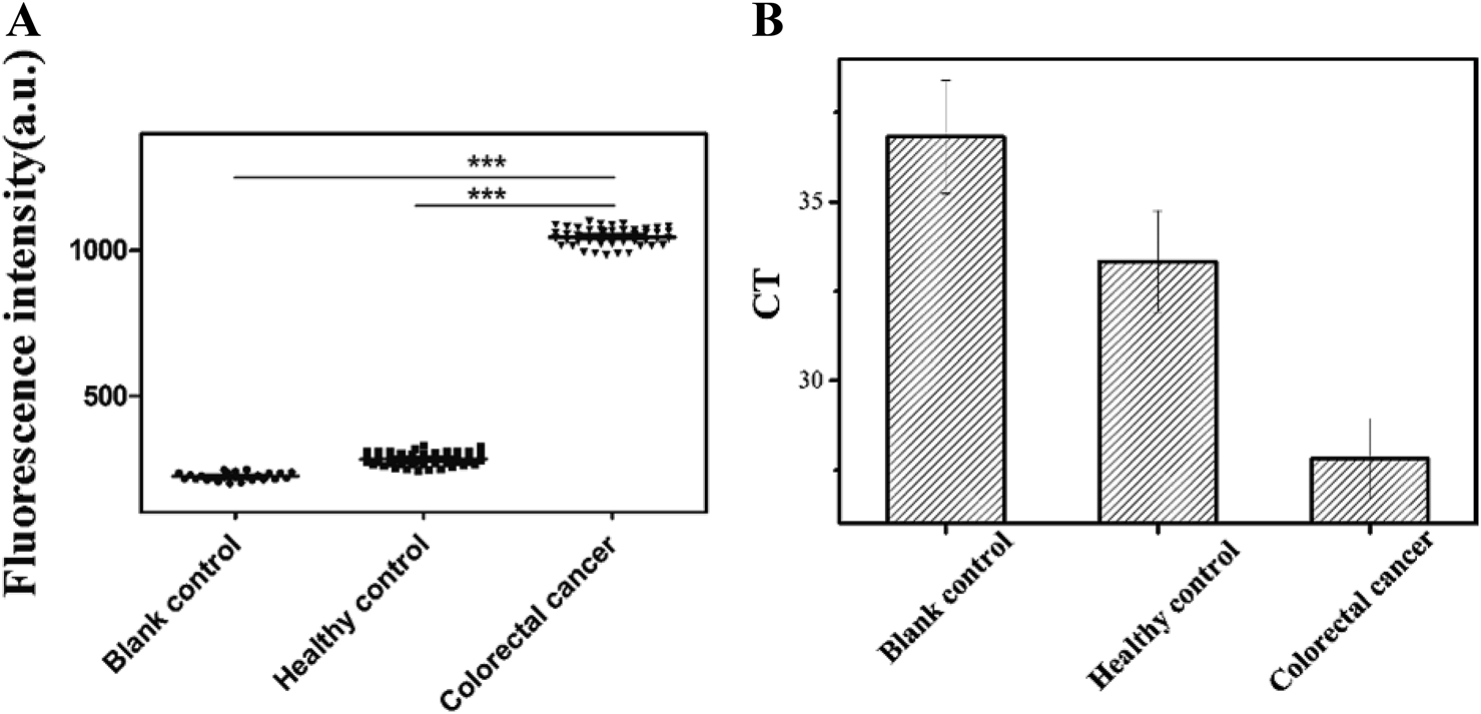
(A) Blood samples assay with aptamers Seq-2 biorecognition ***P < 0.001 compared both the blank control group and the healthy control group indicated that the aptamer Seq-2 possessed high selectivity to the serum from colorectal cancer patients. (B) Mean CT of the aptamers Seq-2 against serum samples of fifty colorectal cancer patients, fifty healthy patients and ultrapure water. The ΔCT value of the aptamers Seq-2 was nearly five indicated the same result.

It was shown clearly in Fig. 5A that both the blank group and the healthy group were significantly different from the colorectal cancer group in regards to the fluorescence intensity. It was also shown in Fig. 5B that the mean CT value of colorectal cancer group was obviously lower than that of healthy group, which illustrated that the Seq-2 nucleic acid aptamer had a high selectivity against the serum from colorectal cancer patients. According to the datas of 52 samples, a respectively bigger amount, it explained that the Seq-2 aptamer could be a potential clinical tool for accurate diagnosis of colorectal cancer through the direct detection of the serum samples of colorectal cancer patients, and also provides a tool for fishing unknown tumor markers in the serum of the colorectal cancer patients.

Due to the lack of blood specimens, testing other cancer serum by Seq-2 as probe will be our next step.

## 4. Discussion

Aptamers, single-stranded nucleic acid molecules that specifically bind a target molecule, are objectively measurable molecular signatures of physiological status that can serve as a tool for disease indicators or drug targets for clinical applications in cancer diagnosis, prognosis, imaging, and therapy. There are many successful study about aptamer-mediated therapy methods on a lot of diseases. Owing to their intrinsic oligonucleotide nature, aptamers have a wide range of targets including proteins, peptides, nucleotides, antibiotics, toxins and small molecules with high affinity and selectivity. There are relatively fewer firsthand studies on serum screening, not to mention colorectal cancer serum. However, as being rich in kinds of proteins playing significant roles in various crucial physiological and pathological processes, serum is the best specimen to vitro diagnosis. Thus, we are determined to screen aptamers against the serum of patients with colorectal cancer aiming to improve their early diagnosis rate and cut the death rate of colorectal cancer.

In this study, we have developed an efficient strategy to screen highly specific nucleic acid aptamers aiming to recognize colorectal cancer as diagnosis tools binding to unknown tumor markers in the serum of the colorectal cancer patients based on a SELEX technology technique assisted by SWCNT.^14–16,33–35^ SWCNT was used to adsorb a serious of ssDNA that did not bind or with weak low affinity with colorectal cancer serum through π–π stacking interaction so that bound ssDNA to SWCNT could be separated thereby the screening efficiency can be improved. Ten rounds of screening were performed to obtain the aptamers with highly specific affinity to tumor markers in the serum of the colorectal cancer patients. Each round of screening consists of several steps including three incubation steps, three elution steps, single PCR amplification step and single dynamic enrichment detection step of the secondary ssDNA sequences, and the second generation high-throughput sequencing analysis of the secondary ssDNA sequences obtained after the final round of screening was performed to determine the enriched nucleic acid aptamer sequences. Therefore, the strategy to screen highly specific nucleic acid aptamers binding to unknown tumor markers in the serum of the colorectal cancer patients based on a SELEX technology technique shows many advantages such as low cost, high efficiency, and accuracy directly detecting with the serum of the colorectal cancer patients as targets.

Moreover, our data demonstrated for the first time that the generated aptamers recognized the colorectal cancer serum with relatively higher affinity and selectivity according to acquired *K*_d_ values in submicromolar range, significantly different fluorescence intensity compared to other aptamer or serum, and relatively larger Δ*C*_t_ values detected by PCR method. Our discovery of aptamers is an advantageous strategy against complex targets particularly in small laboratory. Human serum generally is deemed as a particularly complex target, screening of highly specific nucleic acid aptamers with strong affinity to the serum of the colorectal cancer patients by a SELEX technique with a traditional PCR *in vitro* had not been reported. After 10 rounds, we obtained the four aptamers recognized the serum from colorectal cancer patients with high affinity and selectivity. The *K*_d_ values were all in submicromolar range and relatively larger Δ*C*_t_ values (the difference between the *C*_t_ value of the collected ssDNA binding to the serum from colorectal cancer patients and the *C*_t_ value of the collected ssDNA binding to the serum from the healthy people) were detected by qRT-PCR method. In this study, we used traditional real-time quantitative PCR which accounted for that our screening work don't need high instrument requirements, and it is more helpful for later application comparing to the digital PCR which is difficult to purchase such sophisticated equipment in most laboratories used by Soh's group.^13,39^ Moreover, compared to flow cytometry and elisa for screening,^40–42^ we use PCR more efficiently and conveniently. For the selection of nucleic acid aptamers targeting to the serum from lung cancer patients reported previously by our laboratory, our strategy for selection has been optimized on the basis of anterior work, and the aptamers we obtained have higher affinity under the high efficiency of keeping the number of screening rounds unchanged.^33^ In comparison with purified targets,^43,44^ cell targets,^45–47^ selection against serum targets is more complicated with the difficulty further increasing, although more meaningful for similar to the physiological environment. In addition, with generated aptamers as probes for testing 50 samples, the detection rate reaches over 80% higher than a study than Gupta S's group.^48^ More important, Seq-2, one of the candidate aptamers, which has the highest affinity and the strongest specificity according to the *K*_d_ value and the result of detecting blood samples, exhibits great potential as a nanoprobe for the diagnosis of colorectal cancer. If we want to continue to improve the targeting efficiency and application effect, a small size aptamer is more desirable. Thus, the shortened and optimal aptamers should be explored because not all the nucleotides are necessary for selectively binding tumor markers in the serum and it is our another work to improve it.

In summary, our strategy identifies the four aptamers with highly selective affinity directly screened from colorectal cancer serum and the approach does not need high instrument requirements. Particularly, the aptamer Seq-2 can strongly bind the colorectal cancer serum, less bond non-colon cancer serum and hardly combine the healthy serum. Therefore, aptamer Seq-2 presented enormous potential in exploring of tumor diagnostic kit and fishing unknown tumor markers in serum to reflect colorectal cancer can act as a new molecular tool for accurate clinical diagnosis of colorectal cancer and also can develop a new idea to acquire unknown tumor markers in the serum of the colorectal cancer patients which can tear open another hole for the research on pathogenesis of intestinal cancer.

## 5. Conclutions

In this study, we for the first time screened the new aptamer Seq-2 with highly specific affinity to unknown tumor marker in the serum of the colorectal cancer patients by a SELEX technique and subsequent high-throughput genome sequencing analysis based on the direct utilization of the serum as targeted molecules. In order to maximize the utility of the selected aptamer, we will further study not only the use of selected aptamer to construct sensors for early diagnosis of disease but also a target capture in the blood of colorectal cancer with the generated aptamer, which lays a theoretical foundation for the pathogenesis, drug development, diagnosis and treatment of colorectal cancer thereby providing ideas for precise treatment of colorectal cancer.

## Compliance with ethical standards

The study protocol was approved by the Ethics Committee of the First Hospital of Qinhuangdao, with informed consent signed by all participants.

## Funding

This work was financially supported by the Qinhuangdao Science and Technology Research and Development Plan (No. 201101A132, 201705B024, 201701B044, 201801B035) and by Hebei Science and Technology Research and Development Program Science and Technology Support Program (No. 17272402D).

## Conflicts of interest

The author(s) declared no potential conflicts of interest with respect to there search, authorship, and publication of this article.

## Supplementary Material

RA-009-C9RA04777H-s001
