# Supplementary material for: Selection and preliminary application of a single stranded DNA aptamer targeting colorectal cancer serum
Source: RSC Adv. 2019 Nov 27;9(66):38867–76. doi: 10.1039/c9ra04777h (PMC9075956; doi:10.1039/c9ra04777h)
Supplement: RA-009-C9RA04777H-s001 [file RA-009-C9RA04777H-s001.pdf]

Electronic Supplementary Material (ESI) for RSC Advances.
This journal is © The Royal Society of Chemistry 2019

Selection and preliminary application of a single stranded DNA aptamer targeted colorectal cancer serum

Kun Li[abc]*, Liqing Qi[abc], LiMing Gao[d], Ming Shi[abc], Jian Li[abc], ZhiWei Liu[abc], Lu Zhao[ab]

Table S1. Screening parameters of aptamers against the serum targets of colorectal cancer patients through SELEX technique

Round	Volume of magnetic beads（mL）	Amount of the initial ssDNA library (μg)	Total amount of mixed serum after treatment （μL）	ssDNA library incubation time (min)	Washing buffer	Number of washing	Amount of recovery ssDNA （μg）
1	4	31.40	1000	60	PBS	3	0.52
2	2	28.95	600	60	PBS	5	0.86
3	2	26.49	350	50	PBS+0.02%Tween20	3	0.94
4	1.8	24.68	300	50	PBS+0.02%Tween20	5	1.12
5	1.8	22.87	275	50	PBS+0.05%Tween20	3	1.25
6	1.6	21.54	250	40	PBS+1%Tween20	3	1.48
7	1.6	18.65	225	40	PBS+1%Tween20+ 0.004% SWCNT	5	1.75
8	1.5	15.32	200	40	PBS+1.5%Tween20+ 0.004% SWCNT	3	1.98
9	1.3	12.98	175	30	PBS+2%Tween20 +0.008% SWCNT	5	2.55
10	1	12.69	150	30	PBS+2%Tween20 +0.008% SWCNT	5	2.48

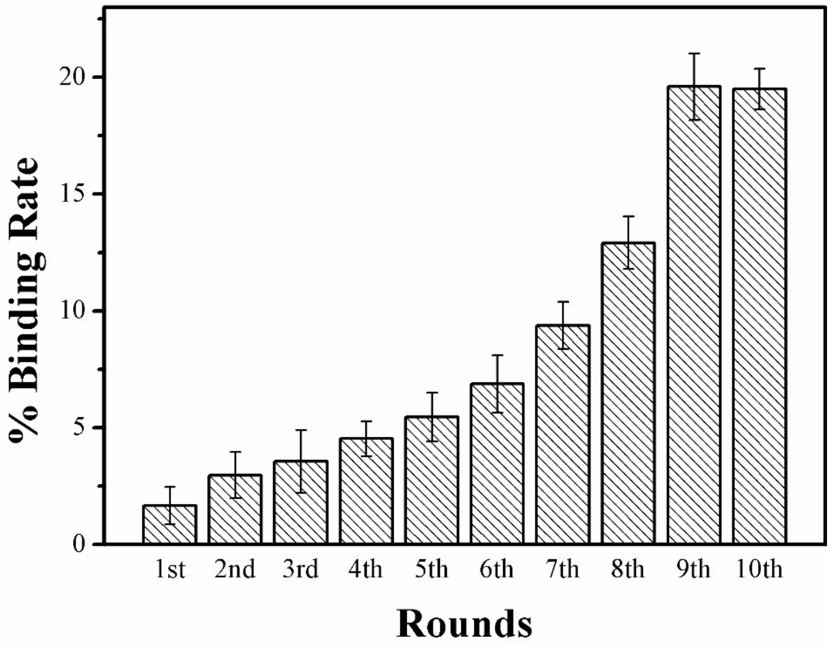

Fig.S1 The recovery rate of each circle during the whole screening process. The amounts of ssDNA recovered in the first 9 rounds were rising up continuously, and there is no evident difference in the 10th round in comparison to the ninth round, which proved the success of the screening. Error bars represent standard deviation from triplicate analysis.

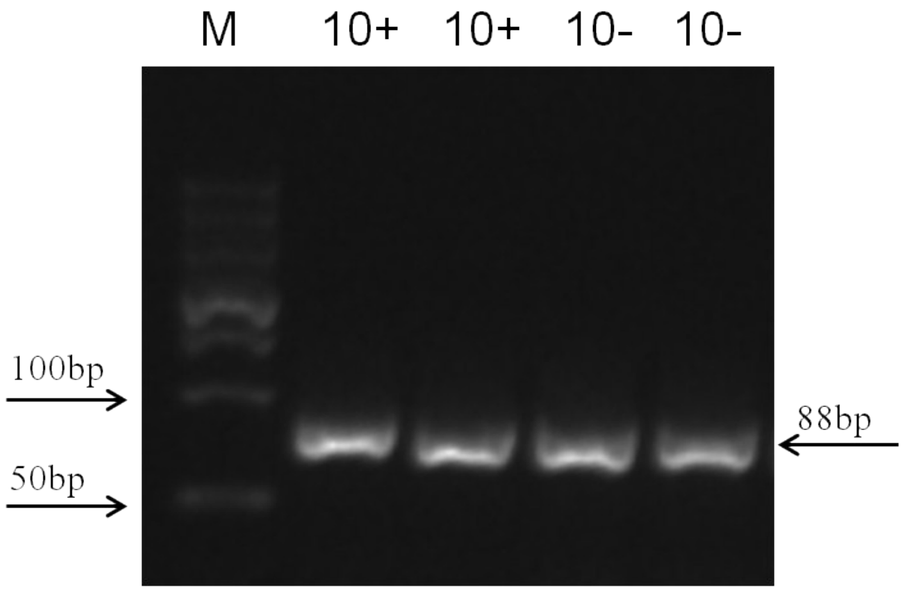

Fig.S2 Gel electrophoresis imaging of PCR amplification products of collected

ssDNA in round 10. The PCR amplification product of collected ssDNA in the colorectal cancer group ran in two parallel 10+ lanes and the PCR amplification product of collected ssDNA in the healthy group ran in two parallel 10- lanes .

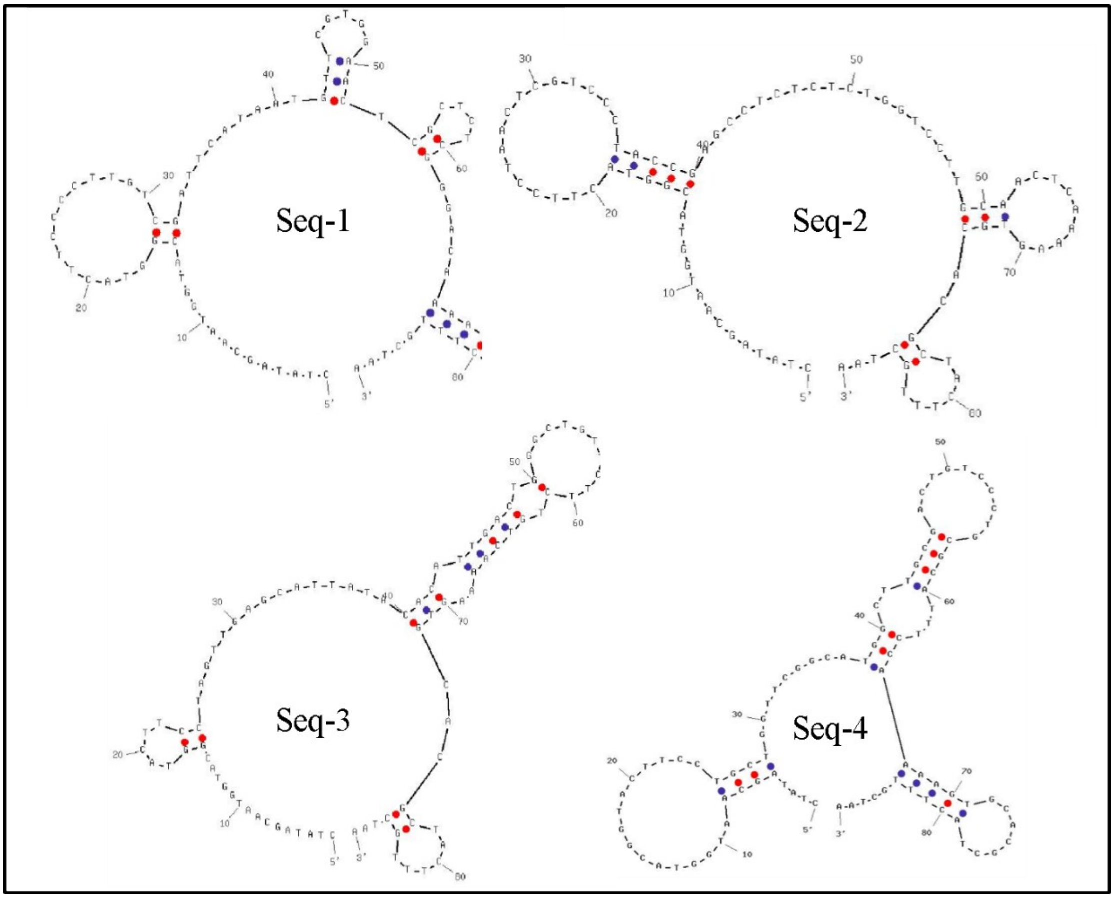

Figure S3. Predicted secondary structure of four candidate aptamers in 37 ^{o}C with IDT.
